# SARS-CoV-2 main protease suppresses type I interferon production by preventing nuclear translocation of phosphorylated IRF3

**DOI:** 10.7150/ijbs.59943

**Published:** 2021-04-10

**Authors:** Sin-Yee Fung, Kam-Leung Siu, Huayue Lin, Man Lung Yeung, Dong-Yan Jin

**Affiliations:** 1 School of Biomedical Sciences, The University of Hong Kong, Pokfulam, Hong Kong; 2 Department of Microbiology, The University of Hong Kong, Pokfulam, Hong Kong; 3 Department of Clinical Microbiology and Infection Control, The University of Hong Kong-Shenzhen Hospital, Shenzhen, China

**Keywords:** SARS-CoV, SARS-CoV-2, NSP5, 3C-like protease, type I interferons, IRF3

## Abstract

Suppression of type I interferon (IFN) response is one pathological outcome of the infection of highly pathogenic human coronaviruses. To effect this, severe acute respiratory syndrome coronavirus (SARS-CoV) and SARS-CoV-2 encode multiple IFN antagonists. In this study, we reported on the IFN antagonism of SARS-CoV-2 main protease NSP5. NSP5 proteins of both SARS-CoV and SARS-CoV-2 counteracted Sendai virus-induced IFN production. NSP5 variants G15S and K90R commonly seen in circulating strains of SARS-CoV-2 retained the IFN-antagonizing property. The suppressive effect of NSP5 on IFN-β gene transcription induced by RIG-I, MAVS, TBK1 and IKKϵ suggested that NSP5 likely acts at a step downstream of IRF3 phosphorylation in the cytoplasm. NSP5 did not influence steady-state expression or phosphorylation of IRF3, suggesting that IRF3, regardless of its phosphorylation state, might not be the substrate of NSP5 protease. However, nuclear translocation of phosphorylated IRF3 was severely compromised in NSP5-expressing cells. Taken together, our work revealed a new mechanism by which NSP5 proteins encoded by SARS-CoV and SARS-CoV-2 antagonize IFN production by retaining phosphorylated IRF3 in the cytoplasm. Our findings have implications in rational design and development of antiviral agents against SARS-CoV-2.

## Introduction

Severe acute respiratory syndrome coronavirus 2 (SARS-CoV-2) is the causative agent of the coronavirus disease 2019 (COVID-19) pandemic 1, 2. This highly pathogenic human coronavirus shares 79.6% nucleotide sequence identity with SARS-CoV 3. Both viruses encode precursor polyproteins pp1a and pp1b, which are proteolytically cleaved by papain-like protease (PL^pro^) NSP3 and main protease (M^pro^) NSP5 into 16 non-structural proteins (NSPs) to assemble the viral replicase complex. Polyprotein cleavage begins from the release of NSP1, NSP2 and NSP3 catalyzed by NSP3 4. Also known as 3C-like protease (3CL^pro^), NSP5 cleaves the remaining proteolytic sites on pp1a and pp1b to release NSP4 to NSP16 5. Assembly of the viral replication complex by NSPs permits *de novo* viral RNA synthesis to drive viral replication and transcription of structural and accessory genes 6.

Highly pathogenic human coronaviruses have developed various countermeasures to circumvent type I interferon (IFN) response 7. Indeed, impaired type I IFN production and signaling have been observed in most severe cases of COVID-19 and in animal models of SARS-CoV-2 infection 8-10. Type I IFN antagonism of multiple SARS-CoV-2 viral proteins have been reported. NSP13 interacts with TBK1 to suppress its phosphorylation 11. ORF6, NSP14 and NSP15 prevents nuclear translocation of IRF3 12, 13. NSP1 binds to 40S ribosomal subunit to shutdown mRNA translation, leading to loss of IFN and IFN-stimulated gene (ISG) expression 14. NSP3 either cleaves ISG15 from IRF3 or cleaves IRF3 directly to attenuate type I IFN production 15, 16. NSP6 counteracts poly(I:C)-induced phosphorylation of IRF3 12. Matrix (M) protein impedes complex formation between RIG-I, MAVS, TRAF3 and TBK1 17. Nucleocapsid (N) protein and ORF8 exert a suppressive effect on Sendai virus (SeV)-induced activation of IFN-β promoter 18-20. ORF9b antagonizes IFN-β production by impeding K63-linked ubiquitination of IKK-γ 21. In addition, ORF3b is another IFN antagonist encoded by some strains of SARS-CoV-2 22, 23.

As described above, NSP5 protease is essential for viral replication 5, 24. The amino acid sequence of NSP5 is divergent among human coronaviruses, but the active sites of cysteine protease are highly conserved 25. Some NSP5 proteases of animal coronaviruses have been implicated in the suppression of type I IFN production and signaling. For example, NSP5 of feline infectious peritonitis virus, which is an alphacoronavirus, is capable of suppressing type I IFN expression through cleavage of IKK-γ 26. NSP5 of porcine deltacoronavirus antagonizes type I IFN signaling through cleavage of STAT2 and DCP1A, which is an ISG with antiviral activity 27, 28. Generally in line with this, cysteine protease activity of SARS-CoV-2 NSP5 is required for the induction of pro-inflammatory response through cleavage of the NLRP12 suppressor of cytokine signaling 16. However, the impact of SARS-CoV-2 NSP5 on type I IFN production remains to be elucidated.

In searching for IFN antagonists among NSPs encoded by highly pathogenic human coronaviruses, we found that NSP5 proteins of both SARS-CoV and SARS-CoV-2 suppress SeV-induced type I IFN production. NSP5 neither cleaves endogenous IRF3, nor inhibits SeV-induced phosphorylation of IRF3. It mitigates type I IFN production by perturbing nuclear translocation of phosphorylated IRF3. Comparison with common polymorphic variants of NSP5 seen in circulating strains of SARS-CoV-2 indicates that their abilities to suppress type I IFN production are unaffected. The conservation of IFN-antagonizing property in NSP5 proteins of highly pathogenic human coronaviruses has implications in viral pathogenesis as well as antiviral and vaccine development.

## Materials and Methods

### Plasmids

Mammalian expression constructs for RIG-IN, MAVS, TBK1, IKKϵ and SARS-CoV ORF6 protein have been described elsewhere 29, 30. Luciferase reporter construct pIFNβ-Luc was kindly provided by Prof. Takashi Fujita (Kyoto University, Japan) 31. cDNAs of SARS-CoV NSP5 and SARS-CoV-2 NSP5 were amplified and cloned from Vero cells infected with SARS-CoV GZ50 strain and SARS-CoV-2 HKU-001a strain, respectively 3, 29, 32. Point mutants of SARS-CoV-2 NSP5 were constructed by Q5® Site-Directed Mutagenesis Kit (New England Biolabs, MA, USA).

### Cell culture, transfection and infection

HEK293, HeLa and A549 cells were grown in DMEM supplemented with 10% FBS and 100U/ml penicillin/ streptomycin (Thermo Fisher Scientific, MA, USA) in a humidified chamber at 37°C, supplemented with 5% CO_2_. HEK293 cells were transfected with Genejuice (MilliporeSigma, MA, USA). HeLa and A549 cells were transfected by Lipofectamine 3000 (Thermo Fisher Scientific). SeV were purchased from American Type Culture Collection (Manassas, VA, USA).

### Luciferase assay and protein analysis

Dual luciferase assay, immunofluorescence and Western blotting were performed as previously described 29, 30. Relative luciferase activity in arbitrary units was calculated by normalizing *Firefly* luciferase activity with *Renilla* luciferase activity. The amounts of IFN-β in conditioned media were measured by VeriKine Human Interferon Beta ELISA Kit (PBL Assay Science, NJ, USA) as per manufacturer's instructions.

Anti-β-tubulin was purchased from MilliporeSigma (Burlington, MA, USA). Mouse anti-V5 was from Thermo Fisher Scientific. Anti-IRF3 was purchased from Santa Cruz Biotechnology (Dallas, TX, USA). Anti-phospho-IRF3 was bought from Cell Signaling Technology (Danvers, MA, USA).

## Results

### Type I IFN antagonism of SARS-CoV-2 NSP5 in comparison with SARS-CoV NSP5

We have performed several rounds of functional screens using expression libraries of all SARS-CoV- and SARS-CoV-2-encoded proteins to search for coronaviral proteins that can suppress type I IFN production. NSP5 proteins of both SARS-CoV and SARS-CoV-2 consistently identified as a novel IFN antagonist in all screens were subjected to further experimental validation reported in this study. NSP5 proteins of SARS-CoV and SARS-CoV-2 share 96% amino acid sequence identity. Notably, the catalytic site residues Cys144 and His41 as well as the Y-X-H motif in NSP5 are highly conserved among all strains of SARS-CoV and SARS-CoV-2. G15S and K90R are two common polymorphic variants of SARS-CoV-2 NSP5 in circulating strains of SARS-CoV-2 33, 34.

**Figure 1:**
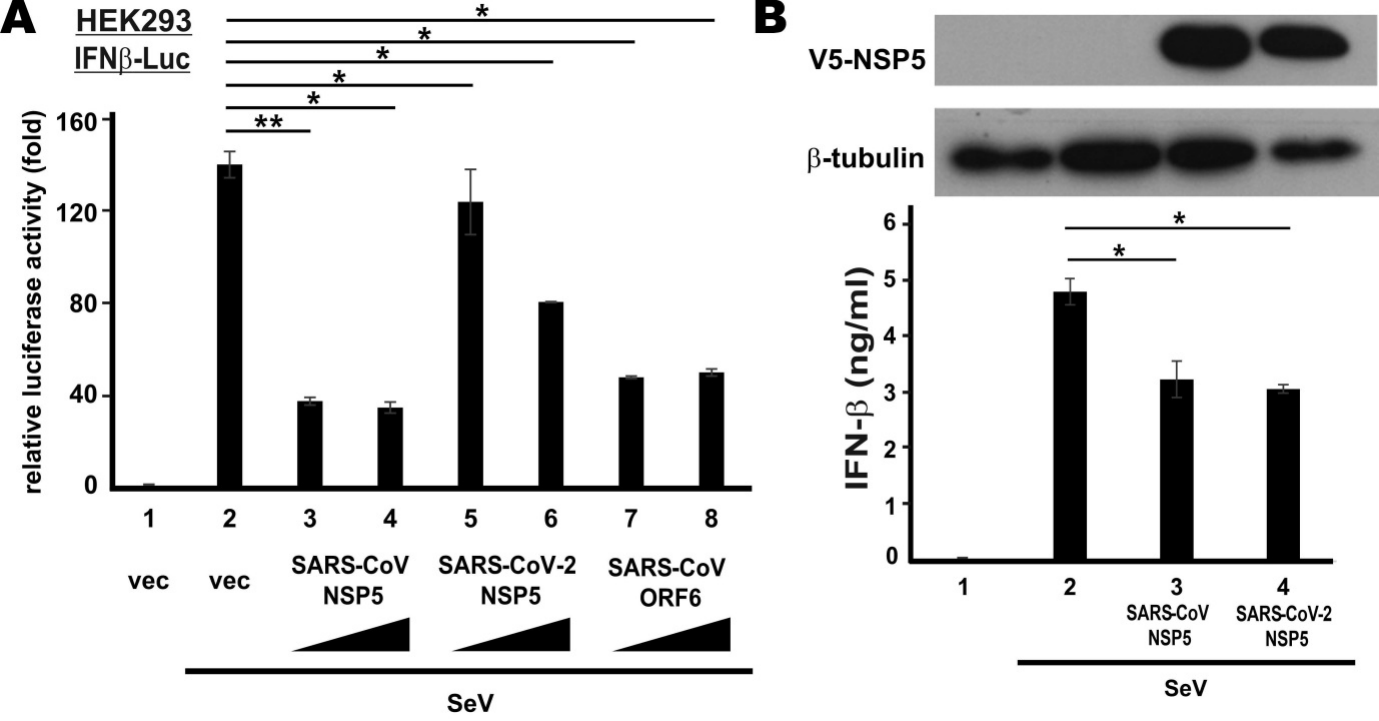
**Suppression of SeV-induced IFN-β expression by SARS-CoV-2 NSP5 versus SARS-CoV NSP5. (A)** Suppression of IFN-β promoter activity in HEK293 cells. Cells were transfected with an IFN-β promoter-driven luciferase reporter construct (IFNβ-Luc) together with increasing doses (400 ng and 600 ng) of expression plasmid for SARS-CoV NSP5, SARS-CoV-2 NSP5 or SARS-CoV ORF6. Transfected cells were infected with 100 hemagglutinating units/ml of SeV at 24 h post transfection. Dual luciferase activity was measured at 24 hpi. **(B)** Suppression of IFN-β secretion in A549 cells. Cells were transfected with expression plasmids for V5-tagged SARS-CoV NSP5 and SARS-CoV-2 NSP5 (V5-NSP5). Transfected cells were infected with SeV at 48 h post transfection. Conditioned media were collected for ELISA analysis of IFN-β at 6 hpi. Expression of V5-NSP5 was detected by Western blotting. The statistical significance between selected samples was evaluated by a one-tailed Student t test for unpaired samples with equal variance. *: P < 0.05. **: P < 0.01.

To verify type I IFN antagonism of SARS-CoV NSP5 and SARS-CoV-2 NSP5, we first checked for their effect on the transcriptional activity of IFN-β promotor in HEK293 cells that have an intact RNA sensing machinery. SARS-CoV ORF6 protein, a known IFN antagonist 35, was included as a positive control. NSP5 was overexpressed in HEK293 cells co-transfected with an IFN-β promotor-driven reporter construct (IFNβ-Luc). SeV, a canonical inducer of type I IFNs, was used to stimulate the activity of IFN-β promoter. SeV infection robustly induced IFN-β promotor activity (Figure 1A, bar 2 compared to 1). Ectopic expression of SARS-CoV NSP5 or SARS-CoV ORF6 was equally effective in blunting SeV-induced activation of IFN-β promoter (Figure 1A, bars 3, 4, 7 and 8 compared to 2). Although the suppressive effect of SARS-CoV-2 NSP5 in this setting was less pronounced, a clear dose dependence was seen (Figure 1A, bars 5 and 6 compared to 2). Next, we validated our findings by assessing the suppressive effect of SARS-CoV NSP5 and SARS-CoV-2 NSP5 on the secretion of IFN-β protein. NSP5 expression was enforced in type II pulmonary epithelial A549 cells. In general agreement with the results from the reporter assays, the amounts of IFN-β protein secreted to the culture media were significantly reduced in cells expressing SARS-CoV NSP5 or SARS-CoV-2 NSP5 (Figure 1B, bar 3 or 4 compared to 2). Thus, both NSP5 proteins function as suppressors of type I IFN production.

### Action point of NSP5 proteins in type I IFN production

**Figure 2:**
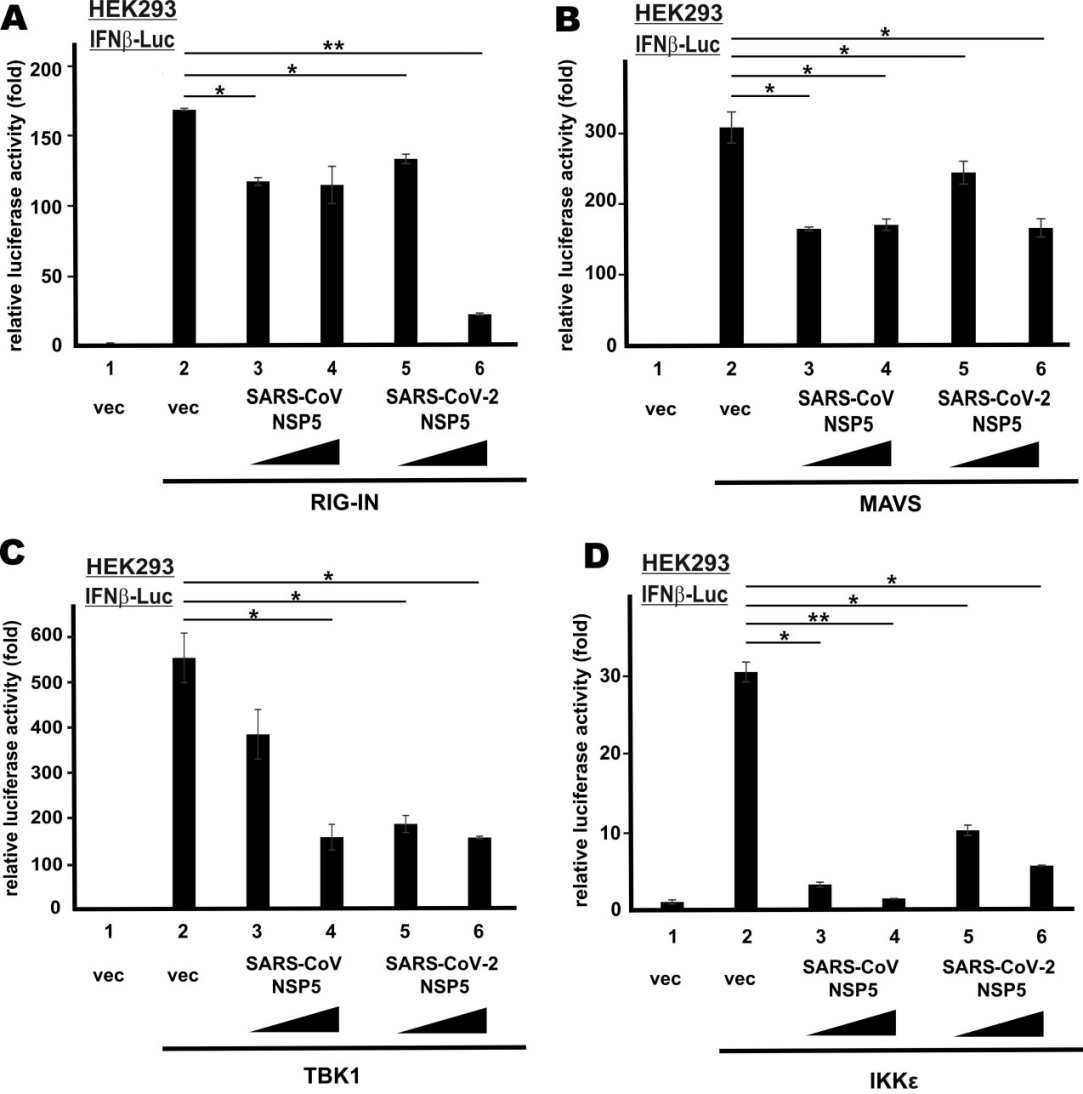
** NSP5 suppresses IFN-β promoter activity downstream of IRF3 phosphorylation.** IFNβ-Luc reporter assays were performed as in Figure 1B, but in place of SeV, the following stimulants were used: **(A)** RIG-IN, **(B)** MAVS, **(C)** TBK1 and **(D)** IKKϵ. Dual luciferase activity was measured 48 h post transfection. The statistical significance between selected samples was evaluated by a one-tailed Student t test for unpaired samples with equal variance. *: P < 0.05. **: P < 0.01.

To define the action point of SARS-CoV NSP5 and SARS-CoV-2 NSP5 in the RNA sensing pathway that leads to IFN-β production, we induced transcriptional activity of IFN-β promoter in HEK293 cells using multiple known activators that govern different steps of the signaling pathway. They included the dominant active form of cytoplasmic RNA sensor RIG-I (RIG-IN), the adaptor protein MAVS, as well as the IRF3 kinases TBK1 and IKKϵ. All four activators robustly stimulated IFN-β promoter activity (Figure 2A-D, bar 2 compared to 1). By and large, SARS-CoV NSP5 and SARS-CoV-2 NSP5 suppressed the stimulatory activity of RIG-IN, MAVS, TBK1 and IKKϵ on IFN-β promoter to similar extent (Figure 2A-D, bars 3-6 compared with 2). Notably, dose dependence was observed in SARS-CoV-2 NSP5-expressing cells in all four experiments. Hence, SARS-CoV NSP5 and SARS-CoV-2 NSP5 counteract RNA-dependent induction of IFN-β expression probably at a step downstream of IRF3 phosphorylation by TBK1 and IKKϵ in the cytoplasm.

### Mechanism by which NSP5 suppresses type I IFN production

**Figure 3:**
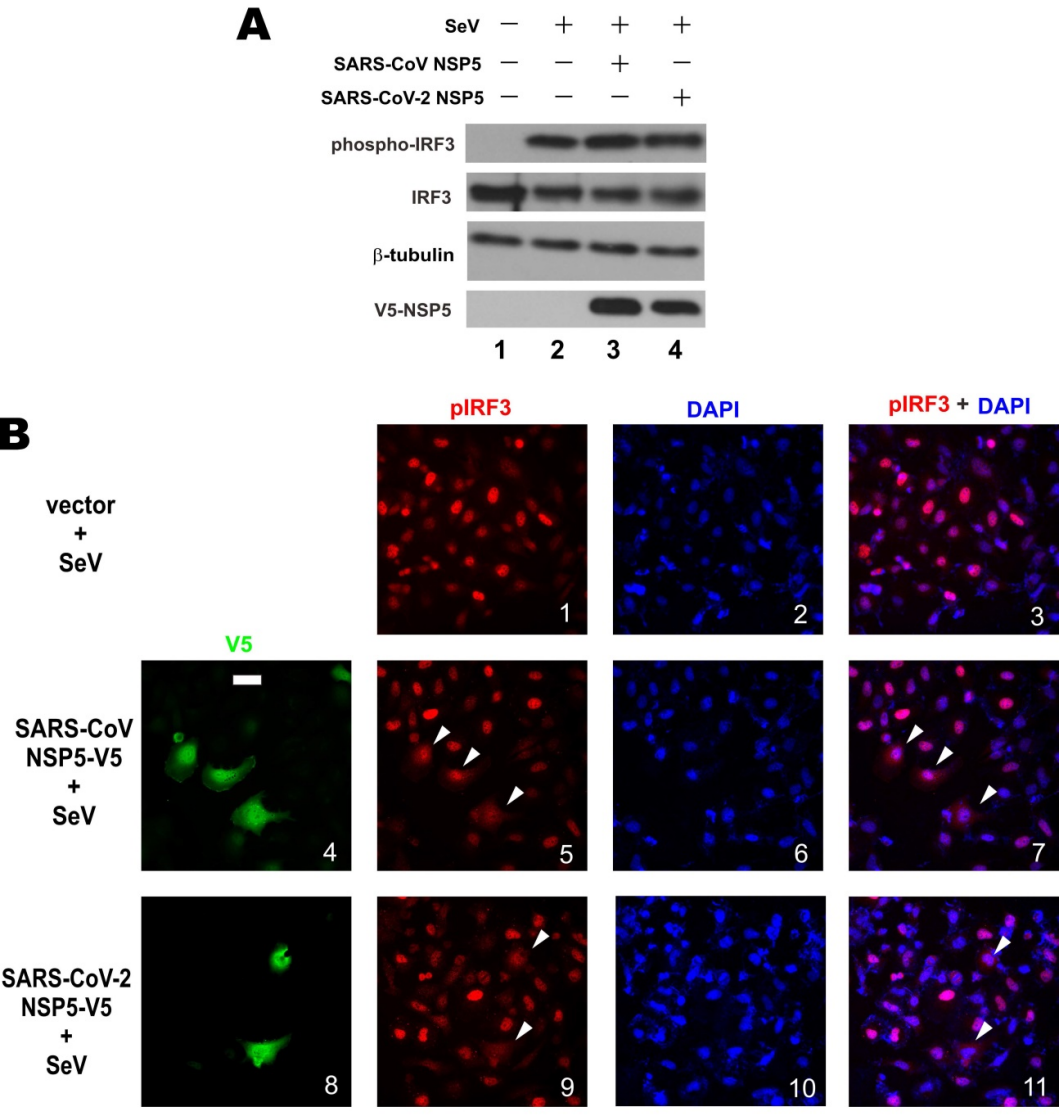
** Prevention of nuclear translocation of phospho-IRF3 by NSP5. (A)** Influence of NSP5 on steady-state expression and phosphorylation of IRF3. A549 cells were transfected with the indicated V5-NSP5 plasmid. At 24h post transfection, cells were stimulated with SeV for 6 h. Cell lysates were analyzed by Western blotting with anti-IRF3, anti-phospho-IRF3, anti-V5 and anti-β-tubulin. **(B)** HeLa cells were transfected with the indicated V5-NSP5 plasmid. At 24 h post transfection, cells were stimulated with SeV for 6 h, and stained for phospho-IRF3 (pIRF3; red signal) and V5-NSP5 (green signal). 4', 6-diamidino-2-phenylindole (DAPI; blue signal) was used to visualized nuclear morphology. The red and blue signals were merged in panels 3, 7 and 11. Bar, 20 µm. Among 100 cells in the slide represented by panel 1, pIRF3 was nuclear in 93 ± 5% of them. Among 50 transfected cells in the slides represented by panels 5 and 9, nuclear localization of pIRF3 was less prominent in 72 ± 4% and 76 ± 6% of them, respectively.

Upon activation, protein kinases TBK1 and IKKϵ are phosphorylated to catalyze the phosphorylation of IRF3 transcription factor. Phosphorylated IRF3 is dimerized and translocated to the nucleus to bind with IRF3 responsive elements and drive type I IFN transcription 36. The 3C-like protease activity of NSP5 and our earlier findings on its action at a point subsequent to IRF3 phosphorylation prompted us to interrogate whether NSP5 might affect the steady-state levels of IRF3 and phosphorylated IRF3. Western blot analysis revealed comparable levels of total IRF3 and phospho-IRF3 in A549 cells overexpressing SARS-CoV NSP5 or SARS-CoV-2 NSP5 compared to mock-transfected cells (Figure 3A, lane 3 or 4 compared to 2), indicating that NSP5 was not influential on stabilization or phosphorylation of endogenous IRF3. Plausibly, the protease activity of NSP5 does not act directly on IRF3 or phospho-IRF3. Considered together with our earlier finding that NSP5 was fully competent in suppressing the IFN-β-inducing activity of IRF3 kinases TBK1 and IKKϵ (Figure 2C and 2D), we reasoned that NSP5 likely suppresses type I IFN expression by affecting the fate or activity of phospho-IRF3.

We next investigated the influence of NSP5 on nuclear translocation of phospho-IRF3. Upon SeV infection, robust nuclear localization of phospho-IRF3 was observed in HeLa cells (Figure 3B, panels 1-3). In stark contrast, nuclear localization of phospho-IRF3 was rarely seen and less prominent in most HeLa cells co-expressing SARS-CoV NSP5 or SARS-CoV-2 NSP5 (Figure 3B, panels 5, 7, 9 and 11, white arrows). Combined, the results indicated that NSP5 antagonizes type I IFN production by preventing nuclear translocation of phospho-IRF3.

### Type I IFN antagonism of NSP5 variants of SARS-CoV-2

**Figure 4:**
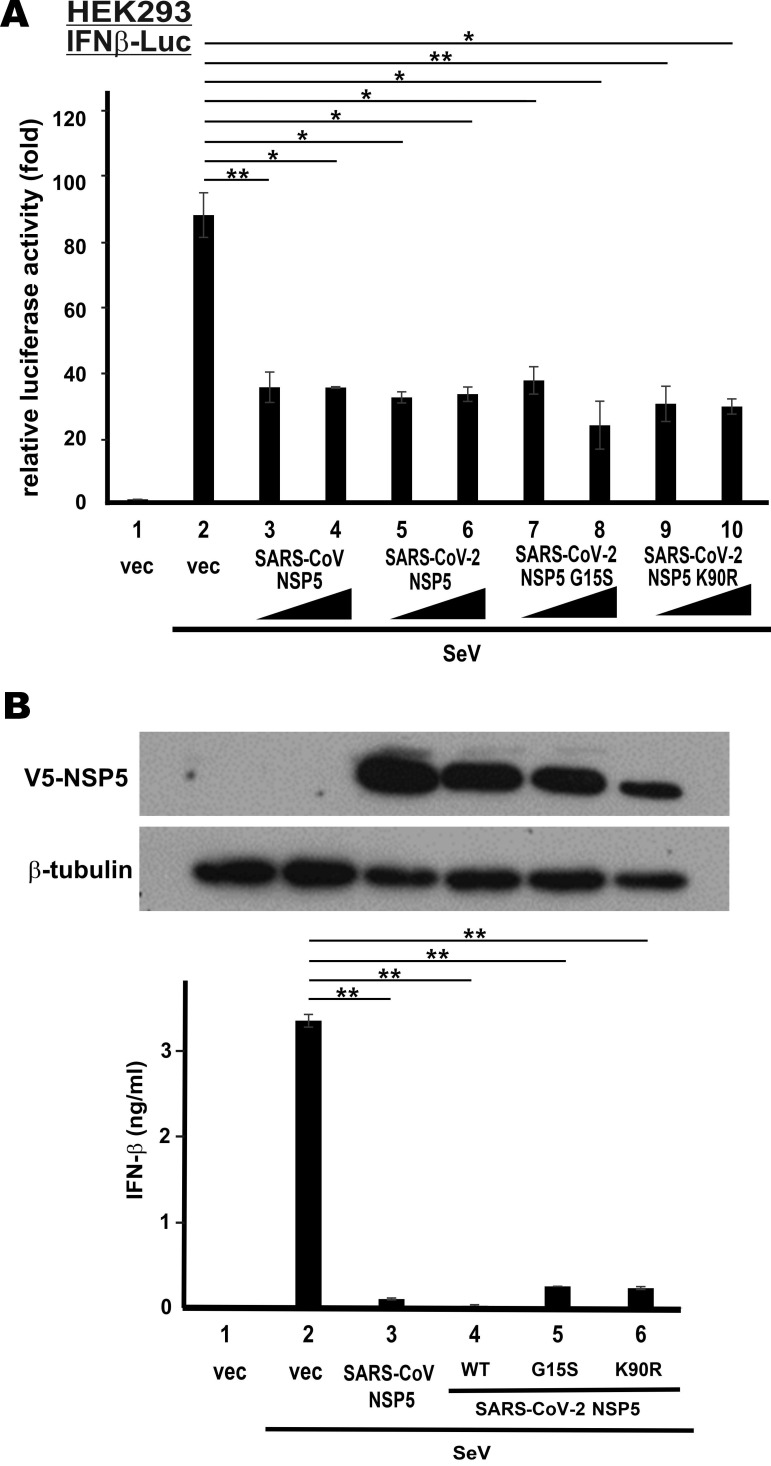
** G15S and K90R polymorphic variants of SARS-CoV-2 NSP5 retains the capability to suppress IFN-β production. (A)** IFNβ-Luc reporter assays.** (B)** IFN-β ELISA. Experiments in Figure 1 were repeated with G15S and K90R variants of SARS-CoV-2 NSP5. Cells were infected with 50 hemagglutinating units/ml of SeV for the IFN-β ELISA experiment. The statistical significance between selected samples was evaluated by a one-tailed Student t test for unpaired samples with equal variance. *: P < 0.05. **: P < 0.01.

Up to date, 22 non‐synonymous substitutions in NSP5 have been discovered in circulating strains of SARS-CoV-2 33. The non-catalytic region of NSP5 is apparently divergent. To determine whether the non-synonymous substitutions might affect type I IFN antagonism of SARS-CoV-2 NSP5, we created its G15S and K90R variants. These two variants were chosen for further analysis since they are frequently seen in circulating strains of SARS-CoV-2 33, 34. Whereas the G15S variant is common in European strains, the K90R variant is more frequently found in Chinese and Icelandic strains 33. Notably, G15S is a novel substitution only seen in SARS-CoV-2, whereas K90R has also been observed in SARS-CoV. We first repeated IFNβ-Luc reporter assays in HEK293 cells with these two variants of SARS-CoV-2 NSP5. Both appeared to be equally competent in the suppression of SeV-induced activation of IFN-β promoter activity, when compared to SARS-CoV NSP5 or SARS-CoV-2 NSP5 (Figure 4A, bars 7-10 compared with 2 and 3-6). Consistent with these results, ELISA measurement of secreted IFN-β in the supernatant of SeV-infected A549 cells verified the suppression of IFN-β production by both variants of SARS-CoV-2 NSP5 (Figure 4B, bars 5-6 compared with 2). Compared to the result in Figure 1B, the suppression was more pronounced when the dose of SeV was halved. Thus, the suppressive activity of the two common NSP5 variants found in circulating strains of SARS-CoV-2 on type I IFN induction remains unchanged.

## Discussion

In this study, we provided the first evidence for suppression of type I IFN production by NSP5 proteins of SARS-CoV and SARS-CoV-2. This suppressive activity remained intact in polymorphic variants of NSP5 found in circulating strains of SARS-CoV-2. NSP5 did not cleave IRF3 and seemingly had no influence on IRF3 phosphorylation. Instead, NSP5 prevented nuclear translocation of phospho-IRF3. Our work provides new mechanistic insight on IFN antagonism of SARS-CoV-2.

Our findings pave the avenue to further mechanistic analysis of the suppression of nuclear translocation of phospho-IRF3 by SARS-CoV-2 NSP5. In the first place, it is crucial to determine the requirement of the 3CL^pro^ activity of NSP5 for its IFN antagonism. Catalytically inactive mutants of NSP5 will prove useful in this analysis. Next, two non-mutually-exclusive models might be tested to shed light on the mechanism of action of NSP5. First, cleavage of nuclear import factors of IRF3 by NSP5 could be tested by a candidate substrate approach. In this regard, SARS-CoV ORF6 and SARS-CoV-2 ORF6 have been shown to target multiple nuclear import factors such as karyopherin, RAE1 and NUP98 to inhibit nuclear translocation of IRF3 and other transcription factors such as STAT1 and STAT2 37-39. It will therefore be of interest to see whether NSP5 might cleave any of these and other nuclear import factors. It will also be intriguing to clarify whether NSP5 might affect IFN signaling by preventing nuclear translocation of other innate immune transducers and effectors such as STAT1 and STAT2. Second, direct binding of NSP5 to IRF3 and phospho-IRF3 should be explored to determine whether they might serve as pseudo-substrate and whether the binding results in cytoplasmic sequestration of phospho-IRF3. Nevertheless, systematic prediction and analysis of cellular proteins that might serve as substrates of NSP5 are warranted.

Main protease is required for coronaviral replication 25. If NSP5 is indeed essential for SARS-CoV-2 replication, catalytically inactive mutations of NSP5 might render SARS-CoV-2 replication-defective. The virus could be rescued by supplying a functional NSP5 in *trans* in the virus packaging cells. The recombinant virus could be further developed and tested as a live but replication-incompetent candidate strain for vaccine development. Similar vaccines have been tested for other viral pathogens such as herpes simplex virus 40. In this regard, whether abrogation of the IFN antagonism of NSP5 might augment propagation of the NSP5-deficient SARS-CoV-2 merits further analysis.

NSP5 protease is an important target for development of antivirals against SASR-CoV-2 25. To this end, a sensitive activity assay for NSP5 41, structural determination of NSP5 42 and proteomic identification of cellular protein substrates of NSP5 43 are pre-requisites for further research and development efforts. Molecular docking 44, *in silico* prediction 45 and compound library screening might also prove useful. Our findings on the type I IFN antagonism of NSP5 raise the possibility that NSP5 inhibitors might sensitize infected cells to the antiviral activity of type I IFNs, providing the rationale for combination therapy using type I IFNs and NSP5 inhibitors. In this connection, a triple combination of IFN- β1, protease inhibitors lopinavir-ritonavir and ribavirin has already been found to be beneficial for treatment of COVID-19 46.
